# TRIM29 mediates lung squamous cell carcinoma cell metastasis by regulating autophagic degradation of E-cadherin

**DOI:** 10.18632/aging.103451

**Published:** 2020-07-08

**Authors:** Weifeng Xu, Beibei Chen, Dianshan Ke, Xiaobing Chen

**Affiliations:** 1 Department of Medical Oncology, The Affiliated Cancer Hospital of Zhengzhou University, Zhengzhou 450008, Henan, P.R. China; 2 Department of Cell Biology, Southern Medical University, Guangzhou 510515, Guangdong, China

**Keywords:** LSCC, TRIM29, EMT, OS, HNBE

## Abstract

Lung squamous cell carcinoma (LSCC) is the most common histological type of primary lung cancer. In this study, we had tested the biological role of TRIM29 in LSCC cells. TRIM29 abundance, the relationships between TRIM29 and E-cadherin and autophagy degradation related proteins in clinical tissues and six cell lines were studied with quantitative real-time PCR test (qRT-PCR) and western blot. TRIM29 overexpression treated HTB-182 cells and knockdown treated NCL-H1915 cells was used for studying cell proliferation, colony formation, migration, invasion, and the expression of epithelial mesenchymal transformation (EMT) associated biomarkers. The relationships between TRIM29 and BECN1 were investigated with western blot. TRIM29 was profoundly overexpressed in LSCC tissues and cells compared with human normal bronchial epithelial cells (HNBE). High TRIM29 expression was closely related to overall survival (OS). TRIM29 overexpression and knockdown affected LSCC activity and the expression of EMT associated biomarkers. TRIM29 can regulate the degradation of E-cadherin and autophagy of LSCC through BECN1 gene, and promote autophagy in HTB-182 and NCL-H1915 cells. Our results revealed that TRIM29 could promote the proliferation, migration, and invasion of LSCC via E-cadherin autophagy degradation. The results are useful for further study in LSCC.

## INTRODUCTION

Primary lung cancer is one of the most common malignant tumors that seriously threaten human health and life. Its morbidity and mortality have increased significantly worldwide, and it has become the leading cause of death from human malignant tumors [1]. In China, primary lung cancer is one of the leading lethal diseases [2]. Lung squamous cell carcinoma is the most common histological type of primary lung cancer. This disease has multiple unique characteristics, including disease progression, local recurrence, distant metastasis, and chemotherapy resistance. Therefore, the overall 5-year survival rates for patients with this disease remain low [3]. Clinically, the prognosis of patients and the formulation of treatment strategies are mainly based on the pathological stage of the disease. However, it is often showed that the prognosis is often significantly different even in patients with the same pathological stage after surgery. Therefore, predicting the prognosis of patients based on the pathological stage alone is relatively low [4]. Therefore, the discovery of molecular markers and mechanisms that can effectively predict lung squamous cell carcinoma can lead to more targeted treatment strategies.

TRIM29 also known as ataxia group D complementary gene (ATDC) is a member of the TRIM protein family. TRIM29 is located on the 11q23 of human chromosome 11. Studies have confirmed that TRIM29 can form homo- or heterodimers during nucleic acid ligation. Meanwhile, this molecule plays a role as a transcriptional regulator during tumorigenesis or differentiation [5–7]. TRIM29 is mainly expressed in the cytoplasm. The molecule is expressed differently in different tissues. We can not detect the expression of this molecule in the heart, brain, kidney, pancreas, condyle, ovary, and small intestine. Meanwhile, it is weakly expressed in the thymus, lung, testis, and prostate. However, it is often expressed in tumor tissues [8]. TRIM29 is involved in multiple physiological processes such as cell proliferation, differentiation, infiltration, migration, and invasion [9, 10]. In cancer study, TRIM29 had been suggested that it was up-regulated expression in multiple cancer types such as pancreatic cancer, gastric cancer, colorectal cancer, lung cancer, bladder cancer, ovarian cancer, endometrial cancer, and multiple myeloma [10]. Kosaka Y et al. suggested that TRIM29 is highly expressed in gastric cancer tissues. The expression of TRIM29 was significantly related to histological grade, tumor size, tumor invasion range and lymph node metastasis [11]. In adenocarcinoma cells, the expression level of TRIM29 is more than 20 times higher than that of normal pancreatic and chronic pancreatitis cells, which suggests that TRIM29 is a good biomarker [12]. Multiple studies have suggested that TRIM29 has important reference value for clinical staging, treatment, and prognosis [13, 14]. Therefore, the molecule is expected to be a target for tumor treatment. However, there is still poor evidence about the role of TRIM29 in lung squamous cell carcinoma.

In this study, we had studied the TRIM29 expression in clinical samples and cell lines. The function of TRIM29 in cell migration and cell proliferation had been probed. In addition, we had demonstrated the detailed molecular mechanisms of TRIM29 molecular in lung squamous cell carcinoma. The results obtained in this study would provide useful information for further study.

## RESULTS

### TRIM29 gene is highly expressed in lung squamous cell carcinoma tissue

**Figure 1:**
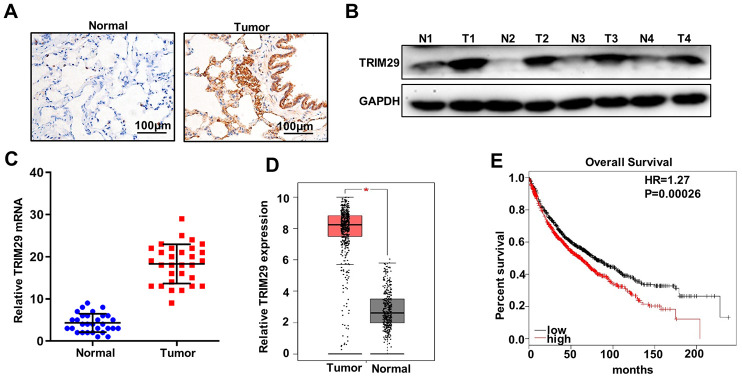
**TRIM29 gene is highly expressed in lung squamous cell carcinoma tissues.** (**A**) Immunohistochemical analysis of normal tissues and lung squamous cell carcinoma tissues. (**B**) Western blot analysis of normal tissues and lung squamous cell carcinoma tissues. N: normal tissues; T: tumor tissues. GAPDH was employed as an internal reference. (**C**) qRT-PCR analysis the TRIM29 mRNA abundance in 30 lung squamous cell carcinoma tissues and paired normal tissues. (**D**) mRNA abundance analysis of TRIM29 gene in GEPIA database (http://gepia.cancer-pku.cn/index.html). (**E**) Validate the relationship between the expression of TRIM29 in lung squamous cell carcinoma and survival in Kaplan-Meier Plotter database (http://www.kmplot.com/). *P<0.05.

Firstly, we examined the expression of TRIM29 in 30 lung squamous cell carcinoma tissues and the paired normal tissues with immunohistochemical staining, western blot, and qRT-PCR analysis. For immunohistochemical staining analysis, the results suggested that TRIM29 expression in lung squamous cell carcinoma tissues was significantly higher than that in the paired normal tissues (Figure 1A). The results of western blot and qRT-PCR analysis indicated that TRIM29 expression in lung squamous cell carcinoma tissues was significantly higher than that in the paired normal tissues (Figure 1B and 1C). Moreover, we evaluated TRIM29 expression in GEPIA database (http://gepia.cancer-pku.cn/index.html). The results revealed that TRIM29 expression in lung squamous cell carcinoma tissues was significantly higher than those in the paired normal tissues (P<0.05) (Figure 1D). In addition, The relationship between TRIM29 expression in lung squamous cell carcinoma and overall survival had been studied with Kaplan-Meier Plotter database (http://www.kmplot.com/). The results suggested that high TRIM29 expression in lung squamous cell carcinoma was significantly related to the short overall survival time (Figure 1E). In summary, TRIM29 expression in lung squamous cell carcinoma tissue was higher than those in the paired normal tissues. Meanwhile, high TRIM29 expression in lung squamous cell carcinoma tissue was closely related to the poor overall survival time.

### TRIM29 promotes metastasis and proliferation of lung squamous cell carcinoma cells *in vitro*

**Figure 2:**
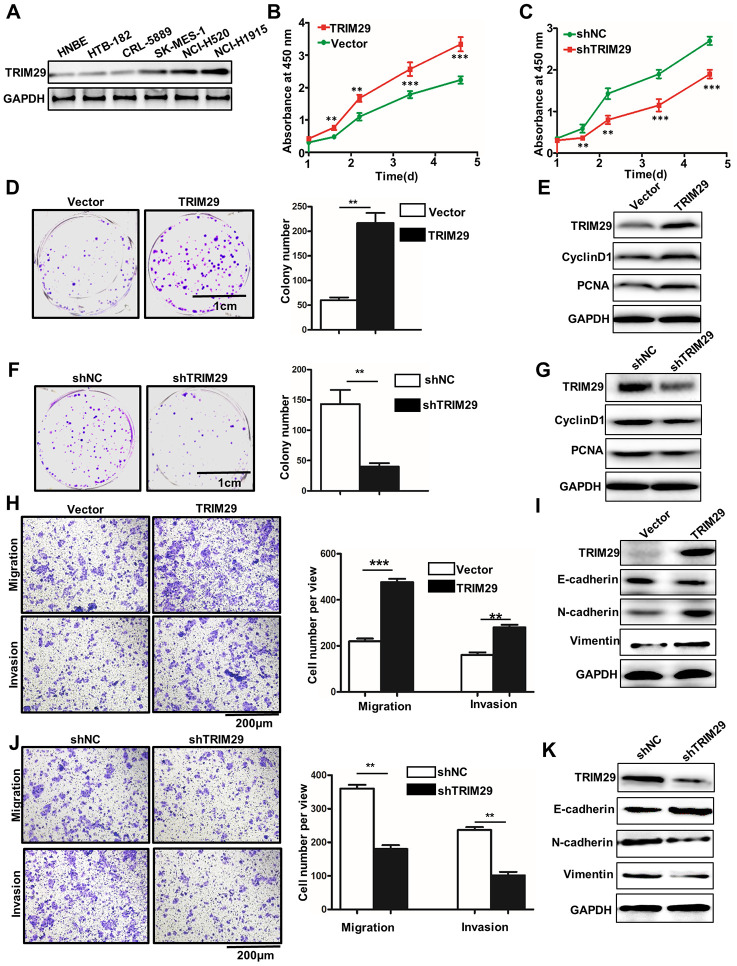
**TRIM29 promotes metastasis and proliferation of lung squamous cell carcinoma cells *in vitro.*** (**A**) Western blot analysis of TRIM29 expression in HNBE, HTB-182, CRL-5889, SK-MES-1, NCL-H520, and NCL-H1915. (**B**) Overexpresson of TRIM29 could significantly promote the proliferation of HTB-182 cells. (**C**) Knockdown of TRIM29 could significantly inhibit the proliferation of NCI-H1915 cells. (**D**) Colony formation analysis of TRIM29 over-expression treated HTB-182 cells. (**E**) Western blot analysis of cell proliferation-related biomarkers expression in TRIM29 over-expression treated HTB-182 cells. (**F**) Colony formation analysis of TRIM29 knockdown treated NCI-H1915 cells. (**G**) Western blot analysis of cell proliferation-related biomarkers expression in TRIM29 knockdown treated NCI-H1915 cells. (**H**) Migration and invasion analysis of TRIM29 over-expression treated HTB-182 cells. (**I**) Western blot analysis of EMT-related biomarkers expression in RIM29 over-expression treated HTB-182 cells. (**J**) Migration and invasion analysis of TRIM29 knockdown treated NCI-H1915 cells. (**K**) Western blot analysis of EMT-related biomarkers expression in knockdown treated NCI-H1915 cells. **P<0.01, ***P<0.001.

In order to further probe the biological function of TRIM29 in lung squamous cell carcinoma cells, we have studied TRIM29 expressions in six selected cell lines, including HNBE, HTB-182, CRL-5889, SK-MES-1, NCL-H520, and NCL-H1915. Western blot analysis of TRIM29 expression in six cell lines indicated that TRIM29 protein expressions were significantly different between each other (Figure 2A). It was notable that TRIM29 protein expression in HTB-182 cells was lower than those in other cell lines. Meanwhile, TRIM29 protein expression in NCL-H1915 cells was higher than those in other cell lines. Therefore, HTB-182 and NCL-H1915 cells were selected to carry out further study. For HTB-182 and NCL-H1915 cells, we performed overexpression and knockdown of TRIM29 treatments, respectively. For cell proliferation analysis, TRIM29 overexpression in HTB-182 cells could significantly promote cell proliferation compared with those in vector treated HTB-182 cells on day 2, 3, 4, 5 (Figure 2A) (P<0.01). However, TRIM29 knockdown in NCL-H1915 cells could significantly reduce cell proliferation compared with those in normal HTB-182 cells on day 2, 3, 4, 5 (Figure 2B and 2C) (P<0.01). Moreover, TRIM29 overexpression in HTB-182 cells could significantly increase cell colony numbers compared with those in vector treated HTB-182 cells (Figure 2D) (P<0.01). Meanwhile, cell proliferation-related biomarkers such as TRIM29, CyclinD1, and PCNA in TRIM29 overexpressed HTB-182 cells were higher than those in vector treated HTB-182 cells (Figure 2E). Furthermore, TRIM29 knockdown in NCL-H1915 cells could significantly decrease cell colony numbers compared with those in normal NCL-H1915 cells (Figure 2F) (P<0.01). Meanwhile, western blot analysis of cell proliferation-related biomarkers showed that TRIM29 knockdown in NCL-H1915 cells was lower than those in vector treated normal NCL-H1915 cells (Figure 2G). In addition, we have also studied the cell migration and invasion in TRIM29 overexpressed HTB-182 cells and TRIM29 knockdown in NCL-H1915 cells. Figure 2H showed that TRIM29 overexpression in HTB-182 cells could significantly increase cell migration and invasion numbers compared with those in vector treated HTB-182 cells (Figure 2H) (P<0.01). TRIM29 overexpression in HTB-182 cells could promote the protein expressions of TRIM29, N-cadherin, and Vimentin. However, TRIM29 overexpression in HTB-182 cells inhibits the protein expressions of N-cadherin (Figure 2I). Those results suggested that TRIM29 overexpression could promote cell migration and invasion in HTB-182 cells. Meanwhile, TRIM29 knockdown in NCL-H1915 cells could significantly reduce cell migration and invasion numbers compared with those in normal NCL-H1915 cells (Figure 2J) (P<0.01). TRIM29 knockdown in NCL-H1915 cells could inhibit the protein expressions of TRIM29, N-cadherin, and Vimentin. However, TRIM29 knockdown in NCL-H1915 cells promote the protein expressions of N-cadherin (Figure 2K).

### TRIM29 regulates autophagy degradation of E-cadherin

**Figure 3:**
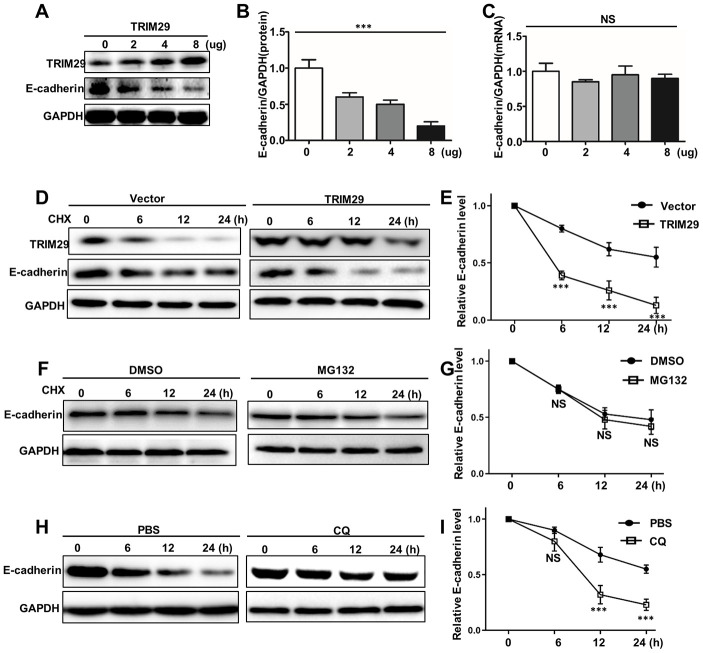
**TRIM29 regulates autophagy degradation of E-cadherin.** (**A**) Western blot analysis of TRIM29 and E-cadherin expression in HTB-182 cells with 0, 2, 4, 8 ug TRIM29 treatment. (**B**) Relative E-cadherin protein expression in HTB-182 cells with 0, 2, 4, 8 ug TRIM29 treatment. (**C**) Relative E-cadherin mRNA expression in HTB-182 cells with 0, 2, 4, 8 ug TRIM29 treatment. (**D**) Western blot analysis of TRIM29 and E-cadherin expression in TRIM29 over-expression treated HTB-182 cells with cyclohexane (CHX) treatment on different time points. (**E**) Relative E-cadherin protein expressions TRIM29 over-expression treated HTB-182 cells with cyclohexane (CHX) treatment on different time points. (**F**) Western blot analysis of TRIM29 and E-cadherin expression in TRIM29 over-expression treated HTB-182 cells with DMSO and MG132 treatment on different time points. (**G**) Relative E-cadherin protein expressions TRIM29 over-expression treated HTB-182 cells with DMSO and MG132 treatment on different time points. (**H**) Western blot analysis of TRIM29 and E-cadherin expression in TRIM29 over-expression treated HTB-182 cells with PBS and CQ treatment on different time points. (**I**) Relative E-cadherin protein expressions TRIM29 over-expression treated HTB-182 cells with PBS and CQ treatment on different time points. ***P<0.001.

Protein stability is mainly affected by proteasome degradation pathways and autophagolysosomal degradation pathways. Therefore, we have identified them separately in this study. In order to probe the potential relationships between TRIM29 and E-cadherin degradation, we performed the western blot and qRT-PCR analysis of TRIM29 and E-cadherin in HTB-182 cells. Figure 3A–3C showed the protein expression and mRNA of TRIM29 and E-cadherin in HTB-182 cells with different TRIM29 dosage treatments. The results suggested that high dosage TRIM29 treatment could reduce E-cadherin protein expression in HTB-182 cells with the dosage-dependent manner. However, no difference of E-cadherin mRNA abundance could be detected in different dosage TRIM29 treatments (Figure 3C). Those results indicated that TRIM29 can reduce the protein level of E-cadherin in a dose-dependent manner without affecting its mRNA levels in HTB-182 cells. Moreover, we have studied the relationships between TRIM29 protein and E-cadherin protein in TRIM29 overexpression HTB-182 cells, which was treated with cycloheximide (CHX). CHX was an agent that could inhibit cellular transcription. Figure 3D and 3E showed that TRIM29 protein could significantly reduce the protein expression of E-cadherin in TRIM29 overexpression HTB-182 cells (P<0.001). MG132 is the inhibitor of proteasome degradation pathway in the cell. In this study, we have employed MG132 (25Um) and DMSO (25Um) to study the E-cadherin protein expression in TRIM29 overexpression HTB-182 cells, which was treated with cycloheximide (CHX). Figure 3F and 3G suggested that no difference of E-cadherin protein expression could be retrieved in TRIM29 overexpression HTB-182 cells. These results suggested that TRIM29 does not affect the proteasome degradation pathway of E-cadherin. In addition, we have further investigated whether TRIM29 affects E-cadherin's autolysosomal degradation pathway. Chloroquine (CQ) is an inhibitor of the autophagolysosomal degradation pathway. In this study, we have employed CQ and PBS to treat TRIM29 overexpression HTB-182 cells, which was treated with cycloheximide (CHX). Figure 3H and 3I suggested that TRIM29 can significantly affect E-cadherin's autolysosomal degradation pathway. E-cadherin protein expression could be significantly reduced in CQ treated HTB-182 cells compared with those in PBS treated HTB-182 cells (P<0.001). In summary, TRIM29 can regulate the autophagy degradation of E-cadherin protein.

### TRIM29 promotes autophagy in lung squamous cell carcinoma

**Figure 4:**
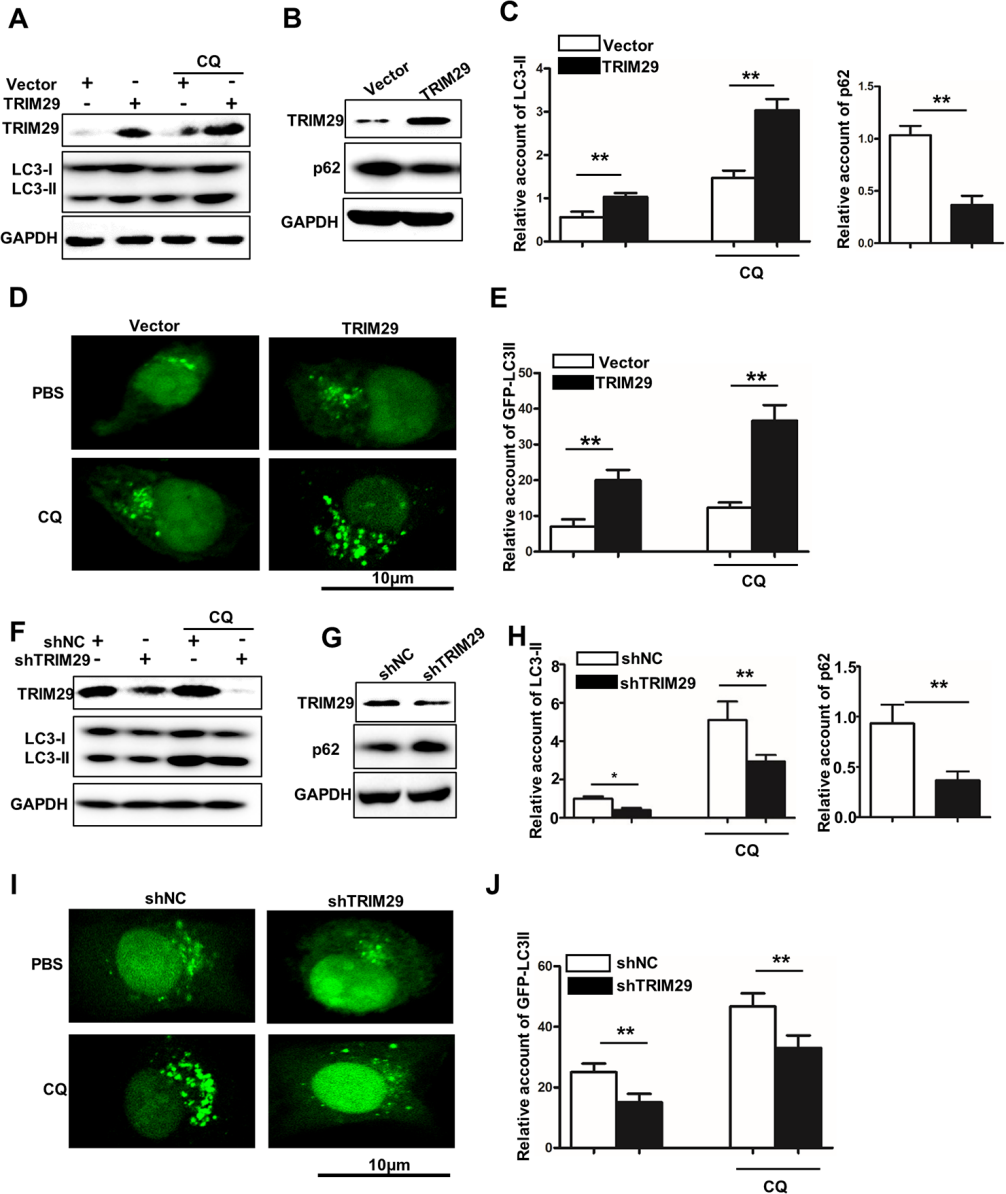
**TRIM29 promotes autophagy in lung squamous cell carcinoma.** (**A**) Western blot analysis of TRIM29, LC3-I, and LC3-II in TRIM29 over-expression treated HTB-182 cells with or without CQ treatment. (**B**) Western blot analysis of TRIM29 and p62 in TRIM29 over-expression treated HTB-182 cells. (**C**) The relative account of LC3-II and p62 in TRIM29 over-expression treated HTB-182 cells. (**D**) confocal analysis of GFP-LC3II expression in TRIM29 over-expression treated HTB-182 with PBS and CQ treatment. (**E**) The relative GFP-LC3II expression in TRIM29 over-expression treated HTB-182 with or without CQ treatment. (**F**) Western blot analysis of TRIM29, LC3-I, and LC3-II in TRIM29 knockdown treated NCI-H1915 cells with or without CQ treatment. (**G**) Western blot analysis of TRIM29 and p62 in TRIM29 knockdown treated NCI-H1915 cells. (**H**) The relative account of LC3-II and p62 in TRIM29 knockdown treated NCI-H1915 cells. (**I**) confocal analysis of GFP-LC3II expression in TRIM29 knockdown treated NCI-H1915 cells with PBS and CQ treatment. (**J**) The relative GFP-LC3II expression in TRIM29 knockdown treated NCI-H1915 cells with or without CQ treatment. **P<0.01.

In this study, we have investigated the potential relationships between TRIM29 expression and autophagy in TRIM29 overexpression HTB-182 cells. Western blot analysis of LC3-I, LC3-II, TRIM29, and p62 suggested that TRIM29 overexpression treatment could significantly increase LC3-II expression and decrease p62 expression in HTB-182 cell, respectively (Figure 4A–4C). We also employed GFP-LC3II to study its expression in HTB-182 cells with different treatments. Figure 4D and 4E showed that LC3-II expression in TRIM29 overexpression HTB-182 cell was significantly higher than those in the vector treated HTB-182 cell. It was notable that GFP-LC3II was distributed with point-like aggregation (Figure 4D). In addition, we have investigated the potential relationships between TRIM29 expression and autophagy in TRIM29 knockdown NCL-H1915 cells. Western blot analysis of LC3-I, LC3-II, TRIM29, and p62 suggested that TRIM29 knockdown treatment could significantly inhibit LC3-II expression and p62 degradation (P<0.001) (Figure 4F–4H). We also employed GFP-LC3II to study its expression in NCL-H1915 cells with different treatments. Figure 4I and 4J showed that LC3-II expression in TRIM29 knockdown NCL-H1915 cells was significantly lower than those in the normal NCL-H1915 cells. It was notable that point-like aggregation of the GFP-LC3II distribution was disappeared in TRIM29 knockdown NCL-H1915 cells (Figure 4E).

### Inhibition of BECN1-induced autophagy can cause the disappearance of TRIM29 related metastasis

**Figure 5:**
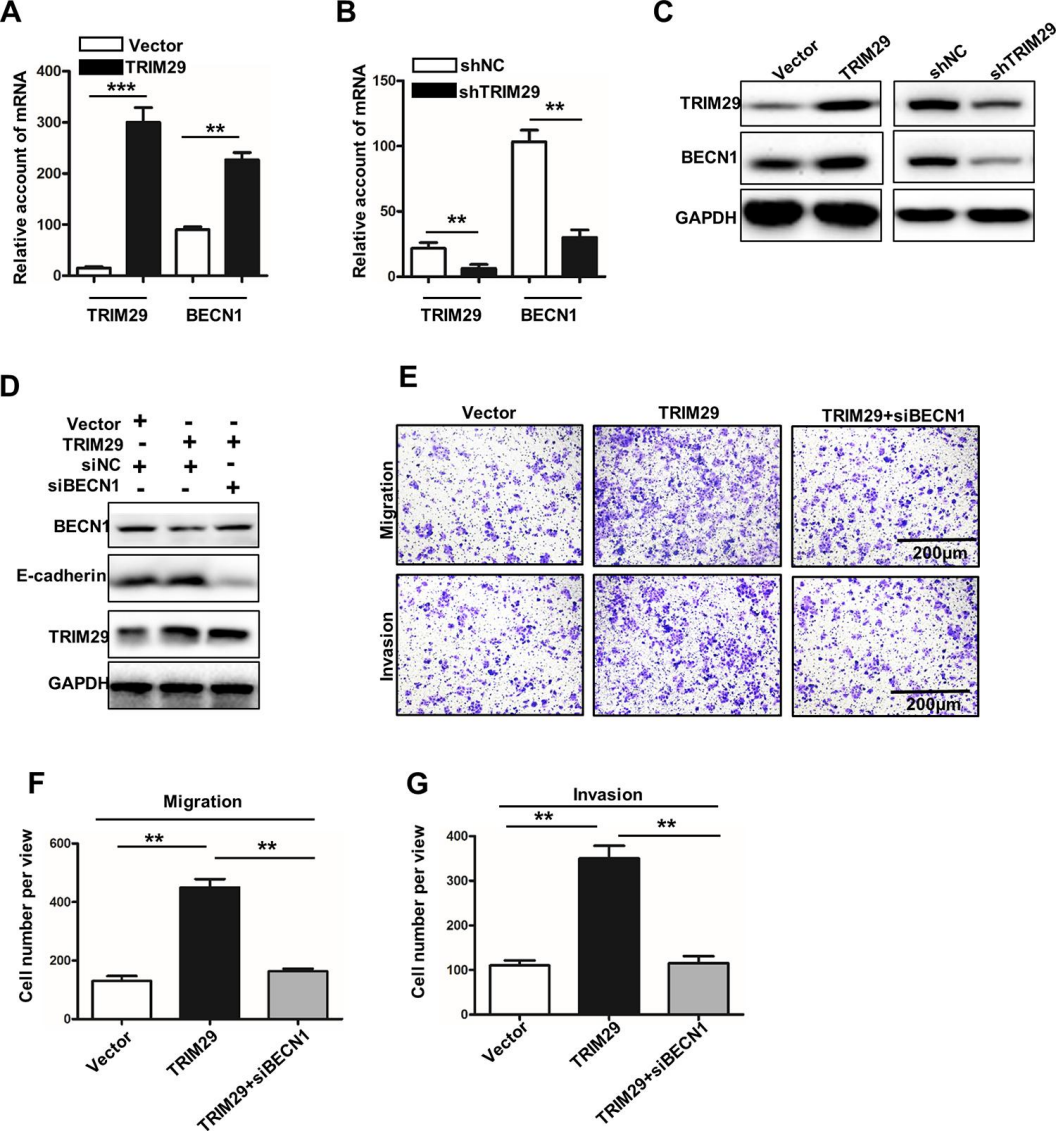
**Inhibition of BECN1-induced autophagy leads to the disappearance of TRIM29-promoting phenotype.** (**A**) qRT-PCR analysis of the TRIM29 and BECN1 expressions in TRIM29 over-expression treated HTB-182 cells. (**B**) qRT-PCR analysis of the TRIM29 and BECN1 expressions in TRIM29 knockdown treated NCI-H1915 cells. (**C**) Western blot analysis of TRIM29 and BECN1 expressions in TRIM29 over-expression treated HTB-182 cells and TRIM29 knockdown treated NCI-H1915 cells. (**D**) Western blot analysis BECN1, E-cadherin, and TRIM29 in TRIM29 over-expression treated HTB-182 cells with BECN1 knockdown treatment. (**E**) Migration and invasion analysis of HTB-182 cells with vector, TRIM29 over-expression, and TRIM29 over-expression+knockdown BECN1 treatment. (**F**) migration analysis of the number of HTB-182 cells with vector, TRIM29 over-expression, and TRIM29 over-expression+knockdown BECN1 treatment. (**G**) Invasion analysis of the number of HTB-182 cells with vector, TRIM29 over-expression, and TRIM29 over-expression+knockdown BECN1 treatment. **P<0.01, ***P<0.001.

In this study, we have studied the BECN1 expression in TRIM29 knockdown NCL-H1915 cells and TRIM29 overexpression HTB-182 cells. Previous studies have shown that high expression of BECN1 in cells can induce autophagy and even autophagic cell death. Meanwhile, the down-regulation of BECN1 expression can significantly reduce cell autophagy response [15]. The BECN1 mRNA expression in TRIM29 knockdown NCL-H1915 cells and TRIM29 overexpression HTB-182 cells was significantly promoted and inhibited (Figure 5A and 5B) (P<0.01). The similar results could be obtained in western blot analysis (Figure 5C). In TRIM29 overexpression HTB-182 cells, we have knocked down the BECN1 gene. Figure 5D showed that E-cadherin's autophagy degradation is inhibited, which was promoted by TRIM29 expression. Moreover, cell migration and invasion analysis showed that BECN1 knockdown treatment in TRIM29 overexpression HTB-182 cells could effectively reduce the number of cell migration and invasion (Figure 5E–5G). In summary, those results mentioned above suggested that knockdown BECN1 can effectively suppress metastasis-promoting phenotype caused by overexpression of TRIM29 in lung squamous cell carcinoma.

### TRIM29 activated BECN1 at the transcription level

**Figure 6:**
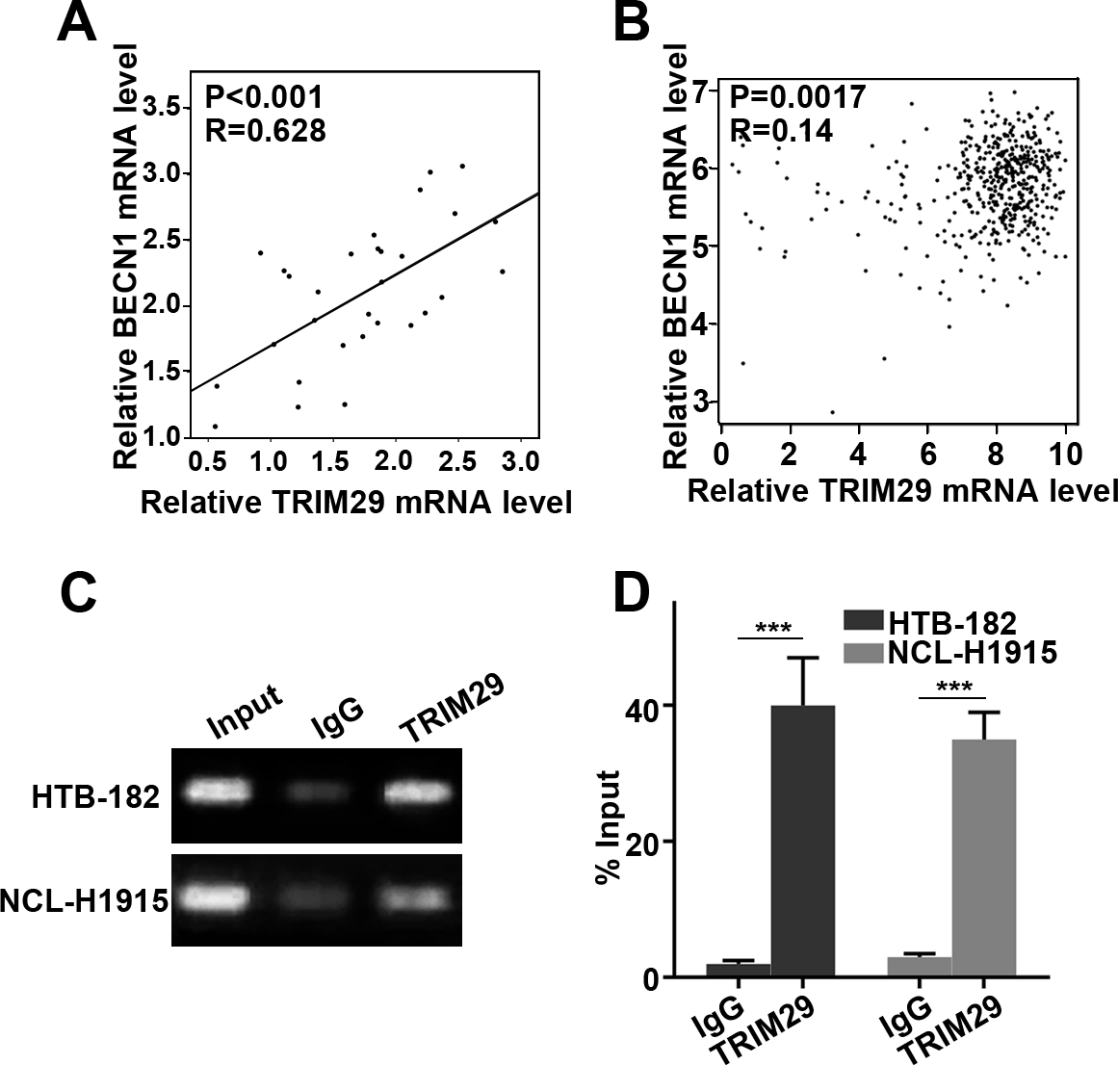
**TRIM29 activated BECN1 at the transcription level.** (**A**) Pearson correlation analysis about the expression of TRIM29 and BECN1 in the tissues. (**B**) Pearson correlation analysis about the expression of TRIM29 and BECN1 through GEPIA data base. (**C**) TRIM29 could combine the promoter area of BECN1 using CHIP assay. (**D**) Measurement of input DNA by qRT-PCR.

To further investigate the regulatory relationship between TRIM29 activated BECN1, we conducted the Pearson correlation analysis about the expression of TRIM29 and BECN1 in the tissues used in this study. A significant positive correlation between the two expression of TRIM29 and BECN1 was observed (Figure 6A). In addition, we further confirmed the expression positive correlation between the TRIM29 and BECN1 using GEPIA data base (Figure 6B). Meanwhile, we found that TRIM29 could combine the promoter area of BECN1 using CHIP assay (Figure 6C, 6D).

## DISCUSSION

Lung cancer ranks first in the mortality rate of male malignant tumors in China. With the advancement of molecular biology and targeted therapy, the efficacy of lung cancer has been improved than before. However, the overall prognosis of the patient remains poor. The high malignancy of lung cancer is closely related to its recurrence and metastasis [2]. Lung squamous cell carcinoma is a common histological type in male non-small cell lung cancer. At present, the basic and clinical research of lung squamous cell carcinoma is still lagging behind, and no specific targeted preparation has been found [16]. Therefore, it is of great clinical significance to study the molecular mechanism of invasion and metastasis of lung squamous cell carcinoma and explore specific targeted therapeutic molecules related to prognosis.

The researcher had cloned TRIM29 for the first time when looking for genes that cause ataxia-telangiectasia (AT) augmentation [17]. Up to now, TRIM29 had been demonstrated with multiple biological functions such as cell proliferation, differentiation, cave death, migration, invasion, and radiation resistance [18]. In tumors, TRIM29 has been suggested to be a potential oncogene and closely related to clinical and prognosis in various tumors [19, 20]. For example, a previous study revealed that immunohistochemical analysis of the expression of TRIM29 showed that high TRIM29 expression reduced overall survival (OS) and disease-free survival. Therefore, high TRIM29 expression may be a potential molecular target for prognostic markers and NSCLC treatment [21]. Sun et al. had employed immunohistochemistry to evaluate the TRIM29 expression n pancreatic ductal carcinoma. Meanwhile, they used a multivariate logistic regression analysis to calculate the association between TRIM29 and clinical characteristics. The results suggested that patients with high TRIM29 expression exhibited shorter overall survival tables and lower relapse-free survival rates. Meanwhile, knocking out TRIM29 gene can inhibit the proliferation, migration, and invasion of pancreatic cancer cells *in vitro* [22]. Moreover, TRIM29 mRNA was significantly increased in patients with nasopharyngeal carcinoma of different pathological types. The expression of TRIM29 is closely related to the depth of tumor invasion, lymph node metastasis, grade and diameter [23]. In this study, our results suggested that TRIM29 was highly expressed in lung squamous cell carcinoma tissues. High TRIM29 expression in lung squamous cell carcinoma tissues was an independent factor of overall survival. The above results were consistent with the identified role of TRIM29 in NSCLC, pancreatic cancer, and gastric cancer. In addition, we have also studied the biological function of TRIM29 in TRIM29 over-expression treated HTB-182 cells and TRIM29 knockdown treated NCI-H1915 cells. The results indicated that TRIM29 expression was closely related to cell proliferation, migration, and invasion, which was adopted to the results report [24].

Epithelial-mesenchymal transition (EMT) refers to the process by which polar epithelial cells are transformed into active mesenchymal cells by the action of extracellular factors [25]. One of the important characteristics of EMT is that the expression of epithelial marker proteins such as E-cadherin is down-regulated and the expression of mesenchymal marker proteins such as vimentin is up-regulated [26]. EMT has been identified to be present in multiple epithelial-derived malignancies [27]. It was notable that E-cadherin (EMT biomarker) was significantly affected in TRIM29 over-expression treated HTB-182 cells and TRIM29 knockdown treated NCI-H1915 cells. TRIM29 can reduce the E-cadherin protein expression without affecting its transcription. Protein stability is mainly affected by proteasome degradation pathways and autophagolysosomal degradation pathways [28]. In this study, our results suggested that TRIM29 does not affect the proteasome degradation pathway of E-cadherin. However, TRIM29 can affect the autophagolysosomal degradation pathway of E-cadherin. Meanwhile, TRIM29 over-expression treated HTB-182 cells and TRIM29 knockdown treated NCI-H1915 cells could affect autophagolysosomal degradation pathway-related proteins expressions such as P62 and LC3. Previous studies have shown that high expression of BECN1 in cells can induce autophagy and even autophagic cell death. Down-regulation of BECN1 expression can significantly reduce cell autophagy response [29–31]. In this study, our results revealed that TRIM29 can regulate autophagy of lung squamous cell carcinoma through the BECN1 gene. Meanwhile, BECN1 knockdown can inhibit the autophagy degradation of E-cadherin promoted by TRIM29 overexpression and cancer cell metastasis. In addition, we speculated that TRIM29 might regulate BECN1 at the transcription level, and proved this using ChIP (Figure 6C, 6D).

In summary, we demonstrated that high TRIM29 expression could be detected in lung squamous cell carcinoma, which was closely related to the OS of patients. TRIM29 could promote proliferation, invasion, and migration in lung squamous cell carcinoma by regulating E-cadherin autophagy degradation. Our results could provide detailed information for further studies in lung squamous cell carcinoma.

## MATERIALS AND METHODS

### Clinical samples and cells

30 lung squamous cell carcinoma samples and their paired normal tissues were collected in the Department of pathology, the Affiliated Cancer Hospital of Zhengzhou University. All experimental protocols were reviewed and approved by the ethics committee of the Affiliated Cancer Hospital of Zhengzhou University. All patients had read and signed the informed consent. The collected tissues were quickly solidified within the fluid nitrogen and stored at -80°C until further study. HNBE, HTB-182, CRL-5889, SK-MES-1, NCL-H520, and NCL-H1915 cells were purchased from ATCC (Virginia, USA). Cells were cultured with RPMI 1640 with 10% (v/v) FBS (Invitrogen, Carlsbad, CA) in a humidified chamber at 5% CO_2_, at 37°C. Double-stranded siRNAs (dsRNA) targeting the TRIM29 Supplementary Figure 1 were synthesized by Sangon Biotech (Shanghai) Co., Ltd. (Shanghai, China). The dsRNA nucleotide sequence for TRIM29 was listed as following: 5’-CAGACACTATATGGAAACT-3’and 3’-GTCTGTATATACCTTTGA-5’. The dsRNA nucleotide sequence for BECN1 siRNA was listed as following:5’-GATCGGTTCCATGTATCGC-3’ and 3’-CTAGCCAAGGTACATATCG-5’. NCI-H1915 Cells were seeded on six-well plates with a density of 5×10^5^ cells per well. DMEM containing 10% FBS without penicillin and streptomycin overnight was used as culture medium. OPTI-MEM serum-free medium (M5650, Sigma Aldrich) and Lipofectamine 2000 reagent (Thermo Fisher Scientific, USA) were used in transfection tests. Final concentration of 100 nM siRNA was introduced in this study. Meanwhile, pEZ-Lv201 Vector was employed to construct the TRIM29 over-expression system in HTB-182 cells. pEZ-Lv201 Vector was used as the negative control in normal HTB-182 cells. Meanwhile, NCL-H1915 cells were used to construct knockdown model of TRIM29 by transfecting sh-TRIM29. The information of designed siRNAs were listed in the Supplementary Figure 1. Lentiviral particles generated with a standardized protocol were used to produce the highly purified plasmids. Endo Fectin-Lenti^TM^ and Titer Boost^TM^ reagents (FulenGen, Guangzhou, China) were used to co-transfect HTB-182 cells. The supernatant was collected after 48 h transfection and stored at-80°C.

### Quantitative real-time polymerase chain (qRT-PCR) analysis

Total RNA was extracted with RNApure Tissue&Cell Kit (CWBio, China). The mRNA expression was detected with Bio-Rad IQ5 system. The real-time PCR reaction contained: 10μL GoldStar Probe Mixture (Low ROX) (CWBio, China), 1μL sense primer (10 nM), 1μL anti-sense primer (10 nM), 2μL cDNA template (10ng), and 6μL H_2_O. The program qRT-PCR was set as following: 95°C, 30 seconds, 40 cycles (95°C, 5 seconds, and 60°C, 10 seconds). 2-ΔΔCt cycle method was used to calculate the relative expression level of mRNAs. GAPDH was employed as the internal control. The results showed in Table 1.

**Table 1:** Sequence of primers for qRT-PCR.

**Gene**	**Forward primer (5’------3’)**	**Reverse primer(5’------3’)**
TRIM29	F-5’-CTGTTCGCGGGCAATGAGT-3’	R-5’-TGCCTTCCATAGAGTCCATGC-3’
GAPDH	F-5’- GGAGCGAGATCCCTCCAAAAT-3’	R-5’- GGCTGTTGTCATACTTCTCATGG-3’
E-cadherin	F-5’-AGATCAGCGACTGTTGTCATC-3’	R-5’-TGCAAGTTGTGACTCATACTTCC-3’
BECN1	F-5’- GTCCCTAGAAGCCAAGCGTAA -3’	R-5’- GGCATATTGCTCATGTTGCTCTG -3’

### MTT

The treated cell suspension (4,000 cells/well) was seeded into 96-well plates. The cells in different groups were cultured in 5% CO_2_/37 °C environment. The proliferation ability of the cells in four groups was detected at the first, second, third, fourth, and fifth day after treatments, respectively. MTT kit was obtained from Sigma-Aldrich (USA). In briefly, 20 ul of MTT solution was added to each well, and cells were cultured for 4 h. The culture medium was carefully removed in each well. 150 ul of dimethyl sulfoxide was added in per well, which was shaken at a low speed for 10 min on a shaker. The absorbance at OD450 was measured after the crystals were thoroughly dissolved. Then, cell proliferation was calculated.

### Colony formation

Cells in different treated groups were inoculated into a 6-well plate at 1000/well, and cultured in 37 °C/5% CO_2_. The cell clone size was observed, and the medium was changed as appropriate according to the medium condition. When macroscopic clones appeared, the culture was terminated. The medium in the well was discarded. The well was washed twice with PBS, and was air-dried. Cells were then fixed with 4% paraformaldehyde for 30 min. After drying, it was stained with 1% crystal violet dye solution for 30 min. subsequently, cell colony formation was observed under an optical microscope.

### Migration and invasion assay

Oris Cell Migration Assay Kit was used to perform cell migration assay (Platypus, USA). Cell invasion was analyzed with EZCell Cell Invasion Assay Kit (Biovision, USA). The detailed steps were strictly followed by the instruction provided by the manufacturer. The number of migrated and invasive cells in the five fields were counted using an inverted microscope (Olympus CKX31, Japan), and the average number was calculated.

### Western blot analysis

Cellular protein in three distinctive groups was confined by 1% PMSF and RIPA lysis buffer (50mM Tris-HCl (pH7.4), 150mM NaCl, 1%NP-40, 0.1% SDS). After reacted with SDS-PAGE test buffer, sodium dodecy lsulfate–polyacrylamide gel electrophoresis was used to perform further examination. At that point, the proteins were exchanged onto a polyvinylidene difluoride layer (Millipore, USA). After being blocked for 1h at room temperature, the layer was brooded with anti-Rabbit TRIM29 (1:1000) (#5182, CST, USA), GAPDH (1:1000) (#2118, CST, USA), E-Cadherin (1:1000) (#31958, CST, USA), LC3-I (1:1000) (#4599, CST, USA), LC3-II (1:1000) (#3868, CST, USA), p62 (1:1000) (#16177, CST, USA), and BECN1(1:1000) (#14717, CST, USA) overnight. Proteins were hatched with the corresponding secondary antibodies for 1 h at room temperature after treated with ECL chemiluminescence kit (Advansta, USA). The bands were observed with GeneGnome 5 (Synoptics Ltd., UK).

### Immunohistochemical staining analysis

The immunohistochemical SP method was used to stain cancer tissue sections. Tissue sections were baked in a 60 °C incubator for 60 min. Then, the tissue sections were subjected to multiple treatments, including immersion in xylene to dewax, gradient alcohol hydration, microwave antigen repair, and 3% hydrogen peroxide treatment. After the goat serum was blocked, an anti-rabbit TRIM29 monoclonal antibody (1: 600) (#5182, CST, USA) was added and incubated at 4°C overnight. We added the universal EnVision complex to the cell culture fluid. A series of test operations were performed, including DAB color development, tap water washing, hematoxylin counterstaining, hydrochloric acid alcohol differentiation, ammonia water back to blue, gradient alcohol dehydration drying, xylene transparent and neutral gum seals. The section was observed under an optical microscope.

### Chromatin immunoprecipitation (ChIP) assay

To investigate the binding between TRIM29 and the region of BECN1 promoters, ChIP assay was conducted using the ChIP-IT Express kit (Diagenode SA, Seraing, Belgium). HTB-182 and NCL-H1915 cells were applied in this assay. The cells were fixed using 1% formaldehyde at room temperature for 10 min. Cross-linking was performed at 37°C for 10 min and was quenched with glycine. Then an antibody against BECN1 (Abcam, UK) was used for immunoprecipitation with IgG (Abcam, UK) as control. The following primers of BECN1 were listed as follows: forward 5'- CAACACTAGGTGCTGGGAATA-3' and reverse 5'- GGCAGAAGACTTGGTGGC-3'.

### Statistical analysis

The values in this study were analyzed by the Student’s t-test. P values < 0.05 were considered to be significant differences between the two data.

## Supplementary Material

Supplementary Figure 1
