# Correction for: TRIM29 mediates lung squamous cell carcinoma cell metastasis by regulating autophagic degradation of E-cadherin

**DOI:** 10.18632/aging.103873

**Published:** 2020-07-30

**Authors:** Weifeng Xu, Beibei Chen, Dianshan Ke, Xiaobing Chen

**Affiliations:** 1 Department of Medical Oncology, The Affiliated Cancer Hospital of Zhengzhou University, Zhengzhou, Henan 450008, P.R. China; 2 Department of Cell Biology, Southern Medical University, Guangzhou, Guangdong 510515, China

**Keywords:** correction

**This article has been corrected:** The corresponding author, Dianshan Ke, requested to change the email. The correct email is given below:

**Correspondence to:** Dianshan Ke; **email: **kds8810@163.com

Original article: Aging. 2020; 13:13488–13501.  . https://doi.org/10.18632/aging.103451

