# Supplementary material for: TRIM29 mediates lung squamous cell carcinoma cell metastasis by regulating autophagic degradation of E-cadherin
Source: Aging (Albany NY). 2020 Jul 8;12(13):13488–501. doi: 10.18632/aging.103451 (PMC7377877; doi:10.18632/aging.103451)
Supplement: Supplementary Figure 1 [file aging-12-103451-s001..pdf]

SUPPLEMENTARY FIGURE

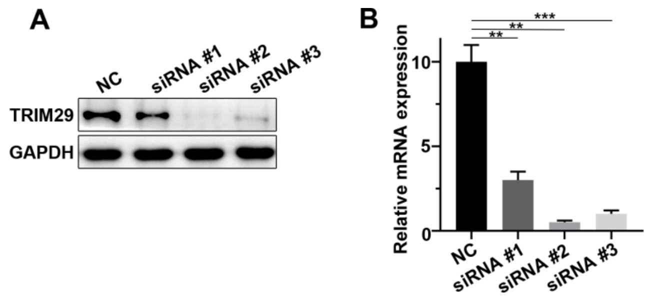

Supplementary Figure 1. The siRNA information of TRIM29. (**A**) Validation of TRIM29 siRNA by western blot; (**B**) Validation of TRIM29 siRNA by qRT-PCR.
